# Social gradient in use of health services and health‐related quality of life of children with attention‐deficit/hyperactivity disorder: A systematic review

**DOI:** 10.1002/jcv2.12170

**Published:** 2023-05-25

**Authors:** Abraham Sevastidis, Sithara Wanni Arachchige Dona, Lisa Gold, Emma Sciberra, David Coghill, Ha Nguyet Dao Le

**Affiliations:** ^1^ Deakin Health Economics School of Health and Social Development Faculty of Health Deakin University Burwood Victoria Australia; ^2^ Murdoch Children's Research Institute Royal Children's Hospital Burwood Victoria Australia; ^3^ School of Psychology Faculty of Health Deakin University Burwood Victoria Australia; ^4^ Departments of Paediatrics and Psychiatry Faculty of Medicine, Dentistry and Health Sciences The University of Melbourne Burwood Victoria Australia; ^5^ The Centre for Social and Early Emotional Development (SEED) Deakin University Burwood Victoria Australia

**Keywords:** ADHD, children, equality, equity, HRQoL, pediatric, service access, service utilisation, SES, well‐being

## Abstract

**Aims:**

ADHD (attention‐deficit/hyperactivity disorder) affects 5% of children on average. Despite the high need to access services for ADHD treatment, not all children with ADHD utilise healthcare services equally. This study aims to systematically synthesise evidence of equity and equality in health service use/costs and health‐related quality of life (HRQoL)/wellbeing of children with ADHD across socioeconomic (SES) classes.

**Methods:**

The literature search was conducted across seven databases (Academic Search complete, MEDLINE Complete, PsycINFO, ERIC, Global Health, CINAHL and EconLit). The search was limited to peer‐reviewed articles published to 23^rd^ January 2023 in English and focused on children. Study quality was assessed using the Critical Appraisal Skills Program (CASP), Joanna Briggs Institute (JBI) and Mixed Methods Appraisal Tool (MMAT) checklists.

**Results:**

25 out of 1207 articles were eligible for inclusion. The results showed that SES was associated with different types of healthcare utilisation. Only three studies were found on HRQoL/well‐being. Children with ADHD from low SES families had lower HRQoL than children from high SES families.

**Conclusion:**

This study found that a social gradient exists in both healthcare service use and children's HRQoL among those with ADHD.

## Graphical abstract

Despite the high need to access services for ADHD treatment, not all children with ADHD utilise healthcare services equally. SES was associated with different types of healthcare utilisation. Only three studies were found on HRQoL/well‐being. Children with ADHD from low SES families had lower HRQoL than children from high SES families. This study found that a social gradient exists in both healthcare service use and children's HRQoL among those with ADHD.

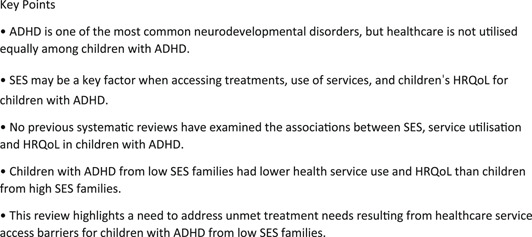

**Key points**

- ADHD is one of the most common neurodevelopmental disorders, but healthcare is not utilised equally among children with ADHD.
- SES may be a key factor when accessing treatments, use of services, and children's HRQoL for children with ADHD.
- No previous systematic reviews have examined the associations between SES, service utilisation and HRQoL in children with ADHD.
- Children with ADHD from low SES families had lower health service use and HRQoL than children from high SES families.
- This review highlights a need to address unmet treatment needs resulting from healthcare service access barriers for children with ADHD from low SES families.

## BACKGROUND

Attention‐deficit/hyperactivity disorder (ADHD) is one of the most common neurodevelopmental disorders, with a prevalence globally estimated at 2%–7% (Sayal et al., 2018). It is increasingly recognised that ADHD symptoms and related difficulties are likely to persist into adulthood (Thelwall et al., 2021). ADHD often co‐occurs with other conditions such as oppositional defiant disorder (ODD), conduct disorder, depression and anxiety (Kadesjö & Gillberg, 2001) and is associated with a range of difficulties such as sustaining injuries, social difficulties, risky behaviours, and lower educational attainment (Enns et al., 2017; Park et al., 2020). Having a child with ADHD is also linked to changes in employment for families, where parents may need to change their working habits to compensate for the increased attention the child with ADHD requires (Azazy et al., 2018). Reduced family employment/income could compound negative consequences, such as reduced access to necessary health services and low medication receipt. Together these factors can reduce children's health‐related quality of life (HRQoL) and contribute to poor long‐term outcomes (D'Amico et al., 2014; Owens, 2020).

ADHD symptoms can manifest in diverse ways depending on developmental stage and this may then be reflected in differences in service usage among children with ADHD at different child ages. For example, in the first year of life, regardless of ADHD status, children tend to use the highest medical services compared to any other year of childhood, which is then reduced with age. However, from the age of 3 years and onwards, hospital‐based service use (e.g., medical and psychiatric services) increases in an almost linear way for children with ADHD (Laugesen et al., 2018). Psychiatric visits are more prevalent in children 15–18 years in general (Lynch et al., 2016). For children with ADHD, emergency psychiatric service utilisation has been shown to increase at age 5–9 years and approaching adolescence (10–14 years) but then seems to reduce by age 15–18 (Lynch et al., 2016).

Multi‐modal approaches to treating ADHD, which combine both psychosocial interventions and medication, are the preferred clinical approach for children and adolescents with ADHD (Subcommittee on Attention‐Deficit/Hyperactivity Disorder and Management, 2011). Interventions such as medication and non‐medication supports have been shown to improve people's lives, by providing healthy symptom management strategies for both parents and children (Faraone et al., 2021). While ADHD medications have shown effectiveness in reducing both inattentive and hyperactivity symptoms of ADHD, improving some cognitive functions (e.g., working memory) (Jensen et al., 2001, 2004) and HRQoL (Coghill et al., 2017), medications sometimes have adverse effects for children with ADHD (e.g., loss of appetite, difficulty sleeping) (Elia & Vetter, 2010).

Non‐medication interventions come in many different forms and vary depending on the age, symptom severity, and particular needs of the child with ADHD. Many national clinical guidelines agree that psychosocial interventions are needed but at different time points of ADHD management and with a focus on improving functioning or HRQoL rather than core symptoms (Coghill et al., 2021). Non‐medication treatments such as cognitive behavioural therapy and parental training have been shown to improve parenting styles and reduce co‐occurring conditions, such as oppositional defiance behaviours in children (Coghill et al., 2021; Faraone et al., 2021). Overall, treatment for ADHD in children needs to be individualised for optimal results (Enns et al., 2017). This should factor in the interplay of treatments, including (but not limited to) a reduction in medication dosage with the introduction of non‐medication therapy and vice versa, depending on the effectiveness of each approach (Coghill et al., 2021; Enns et al., 2017). Despite the various promising treatment options, many families do not have access to psychosocial and pharmacological treatment (DuPaul et al., 2020).

Barriers to access to healthcare services vary depending on the clinical, social, and cultural contexts within patient groups. Barriers to access to healthcare services may reduce the HRQoL of children with ADHD and their families due to unmet treatment needs (Lapresa et al., 2012). A systematic review of international literature reported that common barriers to access include female sex, older age, non‐Anglophonic ethnicity, stigma, and low family socioeconomic status (SES) (Sayal et al., 2018). SES is a measure of a person's or family's social status based on one or more of three factors (income, occupation and education) that determines their economic access to resources and social standpoint in relation to others (Baker, 2014). Social gradients exist for all kinds of health conditions (Veugelers & Yip, 2003) and both physical and mental health conditions in children are impacted by socioeconomic inequalities (Pearce et al., 2019). Access to mental health services, in general, is problematic and inadequate across all ages and social gradients around the world (Saxena et al., 2007). Our systematic review focused on ADHD for several reasons: ADHD is the most common chronic mental health issue in children (Centers for Disease Control and Prevention, 2022; Health & Welfare, 2022); previous studies have reported that ADHD prevalence is negatively associated with SES across countries (Russell et al., 2016; Spencer et al., 2022); moreover, it has been reported that healthcare is not utilised equally among children with ADHD (Sayal et al., 2015); and rates of diagnosis and treatment of ADHD vary greatly between countries (Raman et al., 2018) whilst in many countries ADHD is under diagnosed and under treated (Sayal et al., 2015). Understanding the impact of social gradient on access to services for ADHD is, therefore, important to promote equitable access to all children with ADHD and to minimise the burden of ADHD.

Similarly, SES may be a key factor in children's HRQoL for children with ADHD (Owens, 2020). Treatment options in low SES families can be substantially impacted by treatment costs, combined with the availability and coverage of public health insurance, which varies across countries. For example, in the US, minorities and low SES groups lack access to private health care, and for the same level of need, high SES families have greater access to a wider range of healthcare services for their child with ADHD and co‐occurring disorders, including private and specialist services than low SES families (Husaini et al., 2004). In European countries like Belgium, the out‐of‐pocket cost of healthcare for parents of children with ADHD is six times higher than for children without ADHD (De Ridder & De Graeve, 2006). In contrast, countries such as Sweden have universal health coverage with very low out‐of‐pocket costs for patients, thus families face fewer cost‐related barriers to access to treatment and services in general including ADHD services (Persson et al., 2021). The negative effects of barriers to access on service use and children's wellbeing (i.e., self‐competence and behaviour) in low SES families stem from a systematic lack of resources, whereas families of higher SES can navigate support networks given the higher income of these families, as well as high attainment of health literacy and having social capital to access resources that would increase the use of support services and mitigate detrimental effects on employment, educational and health outcomes in the long term (Owens, 2020).

Any investigation of the association between SES and ADHD must also consider reverse causality, when children's ADHD leads to reduced parental employment and income (Laugesen et al., 2020; Lindly et al., 2021; Ronis et al., 2015), resulting in lower SES. Similarly, parents of children with ADHD can be burdened with stigma, guilt, and exhaustion (Laugesen et al., 2020), which can contribute to unmet treatment needs for children with ADHD, especially in low SES families with limited social and economic support (Owens, 2020). On the other hand, given that ADHD is highly heritable (Buitelaar & Kooij, 2000; Faraone et al., 2021), it is possible that parents of children with ADHD may also have ADHD themselves that might or might not have not been diagnosed. Parental ADHD is associated with negative employment consequences which can lead to lower family SES (Miller et al., 2018; Russell et al., 2014). Co‐occurring parental and childhood ADHD could create a combined impact on lowering family SES.

Research has focused on the social gradient in child's mental health overall, rather than ADHD specifically (Lapresa et al., 2012). Previous reviews on the impact of ADHD on service use/costs or well‐being found a substantial economic impact of ADHD, including poor children's well‐being, substantial costs and productivity losses (Chhibber et al., 2021; Doshi et al., 2012; Lee et al., 2016). However, to the best of our knowledge, there are no previous systematic reviews that have examined the associations between SES, service utilisation and HRQoL in children with ADHD.

It is, therefore, important to explore how SES impacts the use of services and treatment options and children's wellbeing in families of children with ADHD. Better understanding the differences in treatment and wellbeing patterns across the SES spectrum will help policy makers effectively plan services to support these children. This paper aims to explore the associations between family SES and (1) use of services/cost and (2) children's HRQoL/wellbeing among those with ADHD. A synthesis of the existing literature on this topic will aid policy makers in efficiently delivering healthcare services across social groups and improving the wellbeing of children with ADHD.

## METHODS

### Search strategy and study selection

This systematic review adhered to and was reported according to the guidelines in the PRISMA 2020 statement (Page et al., 2021). This review was registered in the PROSPERO database, registration number CRD42022316710 (Sevastidis et al., 2022). A comprehensive literature search was conducted to search for literature published until 23^rd^ January 2023 using seven electronic databases: Academic Search Complete, MEDLINE Complete, PsycINFO, ERIC, Global Health, CINAHL, and EconLit. Primary and secondary research studies published in English (due to limited resources available for translation) were included if they examined inequality across SES in using health services for childhood ADHD or in children's HRQoL in any country. ADHD status could include clinical diagnosis, parents' reporting, or via a research diagnosis. Studies were excluded if they did not address the research questions (e.g., not related to childhood ADHD (children and adolescents aged ≤18 years); not related to HRQoL, service utilisation, or costs), included people older than 18 years of age (without separately reporting results for children or adolescents), or were not published in English. The search terms included broad terms relevant to equity/equality, children, ADHD, service use/costs, and HRQoL (Appendix S1). The search strategy was developed by the primary reviewer (AS) and the senior reviewer (HL) with the consultation of an expert librarian. Hand‐search of the reference lists of the studies included in the full‐text screening was also conducted to locate any additional eligible articles.

Records from the literature search were extracted into Endnote X20 (Clarivate Analytics, Philadelphia, PA, USA). Extracted articles were then screened through a two‐stage process considering the inclusion criteria. Articles were first screened with titles and abstracts by the primary reviewer (AS) and verified by the senior reviewer (HL). Both eligible and inconclusive articles from the first stage were then compiled for full‐text screening. Two reviewers (AS and SWAD) independently screened full texts, and any discrepancies were discussed with the senior reviewer (HL).

### Data extraction

Data extraction was completed by one reviewer (AS) using MS Excel and cross‐checked by another reviewer (SWAD). The information extracted included the author name(s), year of publication, study aims, ADHD diagnosis, study design and duration, location (country), population (age and sample size), outcomes measures, the instrument or tools used, results of outcomes of interest (association of SES with service utilisation/cost/HRQoL), and other factors found to be associated with service utilisation/cost/HRQoL.

### Quality assessment

The quality of cohort studies, randomised controlled trials (RCT) and systematic reviews was appraised using Critical Appraisal Skills Program (CASP) checklists (Critical Appraisal Skills Programme, 2021). The Joanna Briggs Institute (JBI) checklist (Joanna Briggs Institute, 2022) and the Mixed Methods Appraisal Tool (MMAT) checklist (Hong et al., 2018) were used to appraise the quality of cross‐sectional and mixed method studies respectively. CASP checklists have 10–12 questions and are thematically divided into 3 sections, specifically, “Were the results of the study valid?”, “What are the results?” and “Will the results help locally?”. These questions are designed to address the validity, feasibility and applicability of the study being assessed. The JBI checklist comprises questions assessing the validity of the study's methodology and the validity of findings. The main themes of the JBI checklist entail criteria for sample inclusion, detailed descriptions of study subjects, validity/reliability, objectivity, confounding factors and valid outcome measures/statistical analysis applied. The MMAT checklist consists of 5 questions that address the applicability of the methods as well as the quality of the results from the study. Quality appraisal was conducted by two reviewers independently (AS and SWAD), and any discrepancies were discussed and resolved with the senior reviewer (HL).

## RESULTS

### Overview of the studies

**FIGURE 1:**
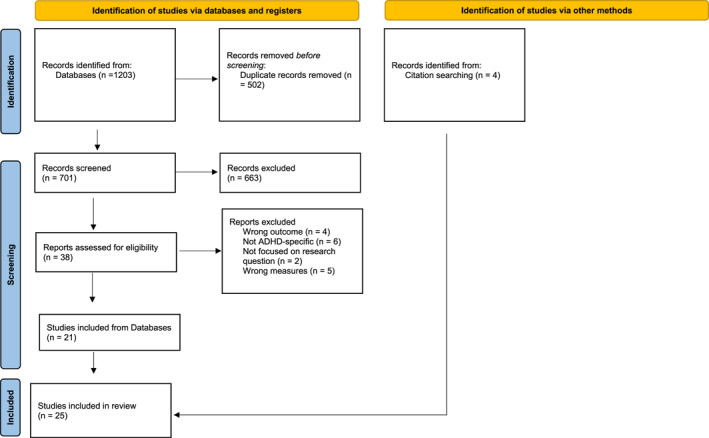
PRISMA diagram.

One thousand two hundred and three (1203) articles from database search and four articles from hand‐search of references were identified. After removing duplicates, of 705 articles from the database and hand‐search of references, 25 met the inclusion criteria for the final synthesis (Figure 1). Table 1 summarises the study characteristics and the findings on the impact of SES on service utilisation and children's HRQoL in those with ADHD. Twenty‐two studies examined socioeconomic differences in service utilisation, and two explored socioeconomic differences in children's HRQoL/wellbeing. Another study was relevant to both themes (see Table 1).

**TABLE 1:** Study characteristics of the included studies.

Author & year	Study type; duration	Country	Population (age in years); sample size	ADHD diagnosis	SES measure	Outcome measures/instrument	Main findings	Other factors
SES and service utilisation/cost								
Countries with a non‐universal healthcare system								
Dejong et al. (2016)	Retrospective Cohort; 2002–2011	US	Children (4–17); *n* = 2591	Clinically diagnosed	Education; income	Annual mental health expenditures and total health expenditures/Survey questionnaire	Higher total health spending for those with a parent with a college degree than those who do not have a college degree	Co‐occurring conditions; children with special health care needs; health insurance; race/ethnicity; geography
Kemper et al. (2013)	Retrospective cohort; 2006–2007	US	Children (7–17); *n* = 5651	Parent‐reported	Education; income	Use of CAM therapies/Survey questionnaire	Higher parental education significantly associated with CAM therapy use (Odd ratio for bachelor's degree or higher vs. high school or less was 3.04, 95% CI 1.76–5.26)	Age; ethnic background; geographical location; co‐occurring conditions; health insurance status; use of prescription medications
Nasol et al. (2019)	Cross sectional; 2014	US	Children (8–17); *n* = 2406	Clinically diagnosed	Income	Unmet need for ADHD treatment/Survey questionnaire	Odds of unmet need in families with the financial impact of ADHD compared to families with no impact was twice higher (*p* < 0.001)	ADHD severity; co‐occurring conditions; primary household language; cultural diversity; parental perceptions of service needs; health insurance
Ronis et al. (2015)	Cross sectional; 2005–2006 and 2009–2010	US	Children (0–18); *n* = 40,242	Parent‐reported	Education; income; employment	Family burden and use of PCMH/Survey questionnaire	RR of 0.68 (95% CI 0.55–0.85) for financial problems for >400 FPL poverty line (statistically significant) RR of 1.03 (95% CI 0.86–1.22) for financial problems for 201–400 FPL RR of 0.96 (95% CI 0.82–1.13) for financial problems for 101–200 FPL RR of 1.60 (95% CI 1.26–2.03) for financial problems for postsecondary education (statistically significant) RR of 1.08 (95% CI 0.84–1.40) for financial problems in high school	ADHD severity; co‐occurring conditions; health insurance; care coordination; assistance with referrals
Bussing et al. (1998)	Cohort; 1995	US	Children (mean age of 9.6); *n* = 499	Parent‐reported	Income	Health service use	Children from low‐SES were less likely than those from high‐SES to see their primary care provider for emotional or behavioural issues (30% vs. 47%, *p* < 0.05)	Health insurance; gender; race/ethnicity
Bussing et al. (2003)	Cohort; 1998–2000	US	Children (mean age 7.6); *n* = 3158	Clinically diagnosed or parent‐reported	Income	ADHD treatments	Poverty predicted lower treatment rates for Children with unmet ADHD care needs	Race/ethnicity; gender
Kendall et al. (2005)	Cross‐sectional; na	US	Parents with at least one child with ADHD (6–18); *n* = 157	Clinically diagnosed	Education; income	Health service use; medication use	Income was not a significant factor in any services used. No significant relationships were found between medication use and income or parental education	Gender; race/ethnicity; single‐mother families
Stevens et al. (2005)	Cross‐sectional; 2000–2001	US	Children (3–18); *n* = 374	Clinically diagnosed	Income	Medication use	Children from higher income families were more likely to use longer‐acting medications than short‐acting.	Age; gender; race/ethnicity; insurance status; region of the country
Cuffe et al. (2009)	Cross‐sectional; 2001	US	Children (4–17); male *n* = 4885; female *n* = 4623	Clinically diagnosed	Education; income	Health service use	Higher family education was associated with mental health care utilisation. Family income was not associated with service use	Health insurance; co‐occurring conditions; urban residence; age
Toomey et al. (2012)	Cross‐sectional; 2007–2008	US	Parents of children (6–18); *n* = 127	Clinically diagnosed	Education; income	Medication discontinuation	No statistical difference in the rate of discontinuation was found by family income, or highest parental education level	Gender; age; race; ADHD severity; co‐occurring conditions; time since diagnosis
Kamimura et al. (2022)	RCT; 2006–2013	US	Children (7–11); *n* = 102	Clinically diagnosed	Income	Medication continuity	No significant association between family hardships and ADHD medication continuity was found. Low‐income were associated with poor ADHD treatment continuity (18 fewer days covered with medications than with high‐income)	Race; age; being female
Lindly et al. (2021)	Narrative literature review	US	Children (14–17)	Clinical & parent‐reported	Income	Family financial problems/Previously published literature	Reduced interventions response or medication use was correlated with lower income/poverty	Races; ethnicity
Countries with a universal healthcare system (mostly with public insurance)								
D'Amico et al. (2014)	Prospective Cohort; 1981–1983 to 2002–2004	UK	Boys (6–7); *n* = 83	Parent & teacher reported	Occupation	Health and social care service use and costs/Questionnaires and CSRI	No association between SES with the cost	Type of the disorder (conduct, hyperactivity or mixed)
Enns et al. (2017)	Retrospective Cohort; 2007–2012	Canada	Children (5–17); *n* = 485 & control *n* = 1884	Clinically diagnosed	Income	Health and social services use (hospital use, ED visits, medication use, medication adherence); and inequities related to SES/Administrative data	At least 0.8 higher medication possession rate in children from high‐income families than low‐income families	Availability of interventions to reduce inequity
Jablonska et al. (2020)	Prospective cohort; 2010–2012	Sweden	Children (6–17); *n* = 276,955	Clinically diagnosed	Education; occupation; income	Utilisation of ADHD medication/Administrative data	Lower SES was associated with increased utilisation of ADHD medication. 2.7% & 1.5% of school children utilised ADHD medication in families with low and high education level of parents, respectively. 2.1% & 1.4% of children utilised medication in families with manual to higher‐level non‐manual occupations. 2.6% & 1.2% children utilised medication in low to high‐disposable‐income families	Single‐parent families; immigrant status
Laugesen et al. (2018)	Retrospective Cohort; 1995–2002 to 2007–2014	Denmark	Children (0–12); *n* = 521,193	Clinically diagnosed	Education; income	Healthcare service use (out‐patient visits, inpatient admissions)/Administrative data	IRR 1.13 (95% CI 1.00–1.27) for increased medical service use in children with ADHD of parents with no/limited education compared with high educational levels 0.82 (95% CI 0.74–0.90) IRR 1.11 (95% CI 1.04–1.19) for increased medical service for family income > Danish Krone 425,000 IRR 1.01 (95% CI 0.94–1.08) for decreased psychiatric service use for family income ≤ Danish Krone 300,000	Low gestational age; geographical location; low birth weight; intellectual disability; co‐occurring conditions; parental psychiatric disorders
Sayal et al. (2015)	Prospective cohort; 2000–2001 to 2005–2006	Germany	Children (4–5); *n* = 81	Parent‐reported	Jarman underprivileged area score	Specialist health service use/DSM‐IV ADHD rating scale; SDQ	Under‐privileged areas were weakly associated with service use	Lack of information; issues with professionals (unhelpful, delays, poor communication); stigma; prior negative experiences; privacy concerns; parental mental health problems
Laugesen et al. (2020)	Multistage mixed method	Denmark	Children (6–12)	Clinically diagnosed	Family composition as a proxy; education; income	Medical service use & everyday life/Interview questions, previously published literature	Being divorced or single was associated with increased psychiatric service use (IRR 1.08 and 1.14)	Co‐occurring conditions; parental mental health issues; disruption of family life; emotional burdens
Tzang et al. (2014)	Cross sectional; NR	Taiwan	Caregivers of children (Mean age of children 9.47 ± 1.97); *n* = 104	Clinically diagnosed	Education	Caregiver's understanding of stimulant usage Use of stimulants/Questionnaire	Maternal higher educational status was less likely to receive correct medical treatment (OR 0.03, 95% CI 0.09–0.98) A significant relationship between the mother's education level and the initial expert consultation for the ADHD diagnosis	Stigmatisation; beliefs; single‐parent family status
Countries with a universal healthcare system (with a combination of public and private insurance)								
Ghosh et al. (2017)	Retrospective Cohort; 2003–2007	Australia	Individuals (0–25); *n* = 725,596	Clinically diagnosed	Relative socioeconomic disadvantage index	Psychostimulant use/Population‐based linked data	Odd ratio of 1.08 (95% CI 1.01–1.15) for having stimulants in the less disadvantaged group than in the least disadvantaged	Gender; country of birth; geography
Miscellaneous								
Green & Langburg (2021)	Systematic literature review	US; Germany; Canada	Children (varied from 2 to 18); varied from 40 to a maximum of 827,396	Clinical & parent reported	Income	Predictors of ADHD psychosocial/Previously published literature	A positive association between family income and ADHD psychosocial service utilisation	Symptom severity; co‐occurring conditions; age; race/ethnicity; gender; parental knowledge of the disorder; insurance coverage; transportation; single‐parent status
Wright et al. (2015)	Systematic literature review	US; UK; Australia; Taiwan; Greece	Children (0–18)	Research diagnosis of ADHD	Insurance and subsidised lunch status as a proxy	Access to care/Previously published literature	Accessing healthcare services was higher in high SES than low SES Lower SES did not appear to lack access to informal support Low SES was an indicator of unmet need, doubling the odds that a child with ADHD would not receive services	Gender; age, ethnicity; geography (urban/rural); stigma
SES and HRQoL/wellbeing								
Countries with a universal healthcare system (with a combination of public and private insurance)								
Van Der Kolk et al. (2014)	Cross sectional; 2010	Netherlands	Parent and children (5–18); *n* = 618	Clinically diagnosed	Education; income	QoL/EQ‐5D and KIDSCREEN‐10 questionnaires	HRQoL utility value 0.83 versus 0.74 for treatment responders versus non‐responders (EQ‐5D HRQoL of treatment responders vs. non‐responders (KINDSCREEN‐10 index 42.24 vs. 40.33) HRQoL of treatment responders versus non‐responders for parent's lower (0.83–0.84) to higher education (0.72–0.73) Parental education was one of the highest predictive factors for treatment response.	Age; co‐occurring conditions; having siblings with ADHD
Lee et al. (2022)	Comparative study; 2020–2021	South Korea	Children (7–12 years old) pre‐COVID *n* = 43, COVID *n* = 36	Clinically diagnosed	Income	QoL	Higher monthly household income (than KRW 20000000) positively predicted the total score of the PedsQL 4.0, (child self‐report)	Caregiver's relation to child; child's age; gender
SES and service utilisation and HRQoL/wellbeing								
Countries with a non‐universal healthcare system								
Owens (2020)	Longitudinal cohort; 1998–1999	US	Children (4–17); *n* = 7330	Clinically diagnosed	Education; income; occupation	Child perceived self‐competence and school behaviours/Social rating scale, self‐description questionnaire	80% of children from both middle and upper SES receiving medication after diagnosis compared to 75% lower SES families Diagnosis had a negative marginal effect on future self‐competence and school behaviour in children from middle and upper SES families	

Abbreviations: ADHD, attention deficit hyperactivity disorder; CAM, complementary alternative medical; CBQ, child behaviour questionnaire; CSRI, client service receipt inventory; DBRS, disruptive behaviour rating scale; DSM‐IV, diagnostic and statistical manual of mental disorders–IV edition; ED, emergency department; EF, executive functions; FPL, federal poverty level; IRR, incidence rate ratio; ML, machine learning; NR, not reported; OR, odd ratio; *r*, cumulative risk; RR, relative risk; SES, socioeconomic status; PCMH, patient centered medical home; SDQ, strengths and difficulties questionnaire; US, united states of america; UK, united kingdom.

### Study characteristics

Among the 25 included studies, two were systematic reviews (Green & Langberg, 2021; Wright et al., 2015) and one was a narrative review (Lindly et al., 2021). The rest were primary studies of which 11 (44% of total) were cohort studies (Bussing et al., 2003; Bussing et al., 1998; D'Amico et al., 2014; deJong et al., 2016; Enns et al., 2017; Ghosh et al., 2017; Jablonska et al., 2020; Kemper et al., 2013; Laugesen et al., 2018; Owens, 2020; Sayal et al., 2015), eight (32%) were cross‐sectional studies (Cuffe et al., 2009; Kendall et al., 2005; Nasol et al., 2019; Ronis et al., 2015; Stevens et al., 2005; Toomey et al., 2012; Tzang et al., 2014; van der Kolk et al., 2014), one (4%) was a mixed method study (Laugesen et al., 2020), one (4%) was a randomised controlled trial (RCT) (Kamimura‐Nishimura et al., 2022), and one (4%) was a comparative study (Lee et al., 2022).

Of 22 primary studies, 12 studies were from the US (54%) (Bussing et al., 1998, 2003; Cuffe et al., 2009; deJong et al., 2016; Kamimura‐Nishimura et al., 2022; Kemper et al., 2013; Kendall et al., 2005; Nasol et al., 2019; Owens, 2020; Ronis et al., 2015; Stevens et al., 2005; Toomey et al., 2012), six studies were from Europe (D'Amico et al., 2014; Jablonska et al., 2020; Laugesen et al., 2020; Laugesen et al., 2018; Sayal et al., 2015; van der Kolk et al., 2014) and there was one study each from Canada (Enns et al., 2017), Taiwan (Tzang et al., 2014), Australia (Ghosh et al., 2017), and South Korea (Lee et al., 2022). These countries were categorised based on the healthcare system as follows (OECD, 2023; OECDiLibrary, 2023; The Commonwealth Fund, 2023):

Countries with a non‐universal healthcare system: the US

Countries with a universal healthcare system (mostly with public insurance)‐ the UK, Sweden, Denmark, Canada, Germany, and Taiwan

Countries with a universal healthcare system (with a combination of public and private insurance): Australia, the Netherlands, and South Korea

The number of participants in these studies ranged from 79 (Lee et al., 2022) to 725,596 participants (Ghosh et al., 2017).

### Quality assessment

All the studies had a focused issue/objective. Overall appraisal of all the cross‐sectional studies was to be “included” according to the CASP checklist. Appendix S2 presents the quality assessment outcomes. Only two cohort studies did not address whether the follow up of subjects was complete enough: one study with more than 20% loss to follow up and another which did not report the loss to follow up (D'Amico et al., 2014; Enns et al., 2017). Of three reviews, one review was a narrative review, but it was also assessed using the tool for systematic reviews (Lindly et al., 2021). None of the reviews conducted quality assessments of their included studies, but all had considered important outcomes and had combined review results reasonably (Green & Langberg, 2021; Lindly et al., 2021; Wright et al., 2015).

## MAIN FINDINGS

### SES and service utilisation/cost

Twenty‐three studies examined the association between SES and service use among children with ADHD (Bussing et al., 2003; Bussing et al., 1998; Cuffe et al., 2009; D'Amico et al., 2014; deJong et al., 2016; Enns et al., 2017; Ghosh et al., 2017; Green & Langberg, 2021; Jablonska et al., 2020; Kamimura‐Nishimura et al., 2022; Kemper et al., 2013; Kendall et al., 2005; Laugesen et al., 2020; Laugesen et al., 2018; Lindly et al., 2021; Nasol et al., 2019; Owens, 2020; Ronis et al., 2015; Sayal et al., 2015; Stevens et al., 2005; Toomey et al., 2012; Tzang et al., 2014; Wright et al., 2015). A broad range of indicators of family SES were used across studies including parental educational status, income, and occupation. Some studies indicated health insurance status or subsidised lunch status (Wright et al., 2015) as a proxy for SES or used health insurance status as an additional economic factor but not as a measure of SES (Green & Langberg, 2021).

#### SES and service utilisation

In the US with a non‐universal healthcare system, most studies found a positive association between SES and service utilisation (Bussing et al., 1998, 2003; Cuffe et al., 2009; deJong et al., 2016; Enns et al., 2017; Jablonska et al., 2020; Kemper et al., 2013; Laugesen et al., 2018; Lindly et al., 2021; Nasol et al., 2019; Owens, 2020; Ronis et al., 2015; Tzang et al., 2014; Wright et al., 2015). Several studies (*n* = 9) found that higher SES was related to a higher uptake of health services, (Bussing et al., 1998, 2003; Cuffe et al., 2009; deJong et al., 2016; Kemper et al., 2013; Lindly et al., 2021; Nasol et al., 2019; Owens, 2020; Wright et al., 2015) and children from low SES families experienced twice as high unmet needs as children from high SES families (Bussing et al., 2003; Nasol et al., 2019). High SES families living in advantaged communities were less stigmatised and thus accessed more health services compared to both low SES families in the same communities and high SES families living in disadvantaged communities (Owens, 2020). Two studies from the US found that income was not a significant factor in health service use (Cuffe et al., 2009; Kendall et al., 2005), but Cuffe et al. (2009) found higher education was associated with mental healthcare utilisation. Regarding the role of SES in the choice of services, high SES families and parents with high education in the US were more likely to use complementary and alternative medical (CAM) therapy (Kemper et al., 2013).

From countries with a universal healthcare system (mostly with public insurance), Sayal et al. (2015) reported that underprivileged areas were weakly associated with service use in Germany. Laugesen et al. (2020), using the family composition as a proxy for SES, found that divorced or single parents (compared to coupled parents) were associated with increased psychiatric service use in children with ADHD in Denmark. In an earlier research conducted in Denmark, the author reported that higher family income had been shown to increase medical service use including psychiatric services, whereas children from families with low or no parental education had more medical services than high educational status (Laugesen et al., 2018). One UK study exploring the long‐term economic burden on the child with ADHD into adulthood found that childhood ADHD, which persists into adulthood, was associated with higher health care and social services utilisation and associated cost, along with criminal justice costs in the long‐term, but there was no association of SES factors with this utilisation and cost (D'Amico et al., 2014).

No studies reported on SES and service utilisation from countries with a universal healthcare system with a combination of public and private insurance.

#### SES and cost of service utilisation

For countries with a non‐universal healthcare system, a US study found that the cost of ADHD treatment was higher for high SES or degree‐holder parents than parents with low SES or lower education qualifications (deJong et al., 2016). For low SES families, the combination of limited employment status, absence of health insurance and costs of care act as barriers to health service access, creating unmet treatment needs for their child with ADHD (Green & Langberg, 2021; Nasol et al., 2019).

No study from countries with a universal healthcare system has reported on the cost of service utilisation.

#### SES and medication use

Studies from the US with a non‐universal healthcare system reported that low SES was associated with increased use of ADHD medication compared to high SES, compared to the positive association between SES and overall service use (Jablonska et al., 2020; Laugesen et al., 2018; Ronis et al., 2015). In the US the ability to access services other than medication is often related to the level of health insurance (either private or public) available to the child's family (Ronis et al., 2015). However, Stevens et al. (2005) found that children from high‐income families were more likely to use longer‐acting medications than short‐acting medication. There are mixed findings in regard to medication continuity and income or education. While Toomey et al. (2012) found no statistical difference in the rate of medication discontinuation by income and education, Kamimura‐Nishimura et al. (2022) reported low‐income was associated with poor medication continuity.

For countries with a universal healthcare system (mostly with public insurance), similar to the US, studies found a negative association between SES and medication use. Jablonska et al. (2020) reported that lower SES in Sweden was associated with higher medication usage for each component of SES (i.e., parents with low education levels, low household income, and occupations (e.g., manual workers)) as well as single‐parent families. Children with ADHD in families with high parental educational attainment in Taiwan were less likely to accept appropriate Methylphenidate treatment, but children who receive Methylphenidate medication treatment were less likely to engage in multiple care‐seeking behaviours (Tzang et al., 2014). On the other hand, Enns et al. (2017) found that SES was positively associated with medication use and adherence in Canada.

#### Other factors and service utilisation

In addition to SES, studies demonstrated a significant association of ADHD service utilisation and costs with other factors. As expected, service use and costs were higher for children with higher severity symptoms and co‐occurring conditions (Cuffe et al., 2009; deJong et al., 2016; Green & Langberg, 2021; Kemper et al., 2013; Laugesen et al., 2018, 2020; Nasol et al., 2019; Ronis et al., 2015; Toomey et al., 2012). Other predictors found by some studies were the child's gender, race/ethnicity, parental knowledge of the condition and its needed services, health insurance status, transportation, immigrant status, rural/urban habitation, family composition (single parent), time since diagnosis, and age (Bussing et al., 1998, 2003; Cuffe et al., 2009; Green & Langberg, 2021; Jablonska et al., 2020; Kamimura‐Nishimura et al., 2022; Kendall et al., 2005; Laugesen et al., 2018; Lindly et al., 2021; Stevens et al., 2005; Toomey et al., 2012; Wright et al., 2015). Younger age was associated with access to ADHD care (Cuffe et al., 2009; Green & Langberg, 2021; Wright et al., 2015). For example, Cuffe et al. (2009) found children aged 9–13‐year‐olds had more medical visits than those aged 14–17‐year‐olds. Kamimura‐Nishimura et al. (2022) reported that older age factored in fewer days of medication use in children with ADHD.

While the lack of knowledge of the availability of specialist services is the most frequently reported barrier to the use of these services, very few studies reported no association between specialist service utilisation and parent's awareness of available services (Sayal et al., 2015; Wright et al., 2015).

### SES and children's HRQoL/wellbeing

For the US with a non‐universal healthcare system, only one study reported a negative association between SES and some domains of HRQoL, specifically self‐competence and school behaviours, among children with ADHD who were not taking medication (Owens, 2020).

For countries with a universal healthcare system (with a combination of public and private insurance), two studies explored the association between SES and children's HRQoL/wellbeing (Owens, 2020; van der Kolk et al., 2014). van der Kolk et al. (2014) used EQ‐5D and KIDSCREEN‐10 questionnaires to assess the HRQoL of children with ADHD in the Netherlands and found a positive association between HRQoL and ADHD treatment response. They found that, according to the EQ‐5D tool, the higher family income was positively associated with the HRQoL of treatment responders. With KIDSCREEN‐10 tool, parents' education was a predictive factor for the HRQoL in treatment responders. Lee et al. (2022) found that in South Korea higher household income positively predicts the total score for PedsQL (pediatric quality of life inventory TM) which assesses the physical, emotional, social and school functioning.

## DISCUSSION

While there are systematic reviews on how ADHD has impacted on either or both service utilisation/costs and HRQoL of children with ADHD (Danckaerts et al., 2010; Lee et al., 2016), to the best of our knowledge, this is the first systematic review that focuses on the association between families' SES on service utilisation/costs and/or children's HRQoL/wellbeing among children with ADHD. Findings from this review indicated that healthcare was not accessed equally across the socioeconomic spectrum, and there are differences in treatment patterns in children with ADHD across SES. Very few research explored the association between SES and children's HRQoL/well‐being which found a positive association between SES and HRQoL or HRQoL domains (i.e. self‐competence and school behaviours) for children with ADHD among treatment responders.

### SES and service utilisation and costs

Our review found a positive association between SES and service utilisation for children with ADHD, however, the findings varied across studies, depending on the country examined. For example, studies from Europe (e.g. Sweden) where there are universal public health insurance systems reported that low SES was associated with increased service utilisation whereas most studies from the US where there is no universal public healthcare system indicated that high SES families, with their private health insurance, have more access to services. Finding that higher SES families access a broader range of services for children with ADHD, including interventions with add‐ons such as CAM and alternative treatments, despite the same level of ADHD treatment needs as low SES families (Kemper et al., 2013) raises concern about the gap in equity for low SES families not only in accessing essential services but also in by‐choice aspects of healthcare interventions. Publicly funded multi‐modal interventions have been shown to reduce inequity by allowing equal access across all socioeconomic classes in Canada (Enns et al., 2017). However, despite being the preferred treatment approach by clinicians, due to the high cost of multi‐model treatment, publicly funded multi‐model treatment is not available in many countries which do not have universal healthcare (e.g. low and middle‐income countries, US) or have high out‐of‐pocket costs of service utilisation (e.g. Australia).

Findings on the association between low SES and increased medication use is consistent with a previous literature review exploring the association between ADHD and service use in youth published 15 years ago (Leslie & Wolraich, 2007). Despite the recommended multi‐model treatment and the needs of children/adolescents with ADHD, medication treatment seems to be more affordable to low SES families. We found that children from low SES families experienced twice as high unmet treatment needs as children from high SES families due to financial difficulties. Specific health programs for children with ADHD, such as CAM therapies, were reported to be less accessible to families of low SES due to the high out‐of‐pocket cost (Kemper et al., 2008). These findings are consistent with the broader literature, which shows that healthcare is utilised differently across SES groups, where public healthcare is accessed more often by poorer populations due to low or no out‐of‐pocket expenses, whereas wealthier populations often access private and specialist healthcare services with a high out‐of‐pocket cost (Bakar et al., 2019). Differences in insurance coverage and the ability to activate monetary capital between SES groups were reasons for discrepancies in healthcare utilisation (Husaini et al., 2004).

Although we did not specifically explore the association between SES and educational services, and thus, did not find any study that examined the association between SES and educational services, the negative impact of ADHD on functioning at school including academic attainment is well‐documented in the literature (Lee et al., 2016; Shaw et al., 2012). However, a substantial proportion of children and adolescents with ADHD do not have access to educational support services due to barriers, such as limited budgets in schools, demands for academic accountability, and limited training for teachers regarding behaviour modification (Leslie & Wolraich, 2007; Zendarski et al., 2020). It is, therefore, critical for future research to investigate the association between SES and educational services and the barriers to accessing these services.

In addition to SES, there are a number of other factors that were associated with service utilisation. Specifically, needs factors including the presence of co‐occurring conditions and severity of ADHD contributed to the uptake level of services among children with ADHD (deJong et al., 2016; Green & Langberg, 2021; Kemper et al., 2013; Laugesen et al., 2018, 2020; Nasol et al., 2019; Ronis et al., 2015). Furthermore, disposal factors such as child age and gender, race/ethnicity, geographical location, parental knowledge of the condition and its needed services, immigrant status, single parent, and cultural perspectives also play a role in service utilisation for children with ADHD (Green & Langberg, 2021; Jablonska et al., 2020; Laugesen et al., 2018; Lindly et al., 2021; Wright et al., 2015). Interventions for children with ADHD should take into account these factors to equitably improve accessibility and uptake of ADHD treatment.

### SES and children's HRQoL/wellbeing

While there is literature on the negative impacts of ADHD on children's HRQoL (Lloyd et al., 2011; Nucifora & Walker, 2021), the association between SES with HRQoL for children with ADHD has largely been unexplored. We found only two studies that investigated this topic, both of which found an impact of SES on HRQoL of children with ADHD: Owens (2020) reported a negative association between SES and perceived self‐competence and school behaviours among medicated children with and van der Kolk et al. (2014) reported that pharmacological treatment with most common methylphenidate or atomoxetine has shown to improve children's HRQoL and that parent's education had a positive association with the treatment response. This finding is consistent with the literature on the effectiveness of ADHD treatment (Caye et al., 2019; King et al., 2006). The limited evidence on the association between SES and the HRQoL in children with ADHD suggests that future research is needed in this area. Given that HRQoL has been increasingly valued as a key factor to understand the impact of health conditions in children and included as an outcome measure of ADHD treatment (Adamo et al., 2015), it is important to understand the association between SES and children's HRQoL. This understanding will help clinicians and population health experts to plan effective treatment/interventions that can also improve children's well‐being in addition to the improvement of healthcare systems.

### Strengths and limitations

The strengths of this study include the extensive literature search, and rigorous assessment of the available current literature. However, as limitations, non‐English literature was not included. Secondly, the search may have missed some literature as there is a possibility that some search terms may not have been included in the search strategy. Thirdly, this review is focused on children with ADHD, and the findings here may not apply more generally to children with other disorders.

### Implications for policy and future research

Given ADHD treatment can improve outcomes for children with ADHD (Shaw et al., 2012), understanding the barriers to service utilisation to develop strategies to improve service access and utilisation for ADHD is critical. Our findings highlight a need to address the unmet treatment needs for children with ADHD from low SES families. It is important to recognise the barriers to accessing assessment and multimodal treatment and‐support for ADHD (Enns et al., 2017; Green & Langberg, 2021; Kemper et al., 2013; Owens, 2020; Ronis et al., 2015). This review also highlights that children with ADHD from low SES families need stronger financial support to increase access to services. Given the importance of parental knowledge of ADHD and their awareness of treatment and support services, strategies to improve parental awareness of ADHD, its long‐term outcomes and available services are essential. Furthermore, support for at‐risk groups such as children from immigrant families, single‐parent families, or from regional/remote areas would also be needed to equitably improve access to services or treatment for these children.

## CONCLUSION

This systematic review found a positive association between SES and health service utilisation and HRQoL in children with ADHD. The financial barrier is one of the main factors affecting health service utilisation for children with ADHD. This review highlights a need to address unmet treatment needs resulting from healthcare service access barriers, especially for children with ADHD from low SES families. Policy‐makers should also consider reducing the disparities in the provision and accessibility to healthcare services to children with ADHD from all social gradients to improve HRQoL for children with ADHD. Given the scarcity of research exploring the association between SES and children's well‐being/educational services, future research exploring these topics is warranted.

## AUTHOR CONTRIBUTIONS

**Abraham Sevastidis**: Conceptualisation; Data curation; Formal analysis; Methodology; Writing – original draft. **Sithara Wanni Arachchige Dona**: Data curation; Formal analysis; Methodology; Writing – original draft; Writing – review & editing. **Lisa Gold**: Supervision; Writing – review & editing. **Emma Sciberras**: Writing – review & editing. **David Coghill**: Writing – review & editing. **Ha Nguyet Dao Le**: Conceptualisation; Funding acquisition; Methodology; Supervision; Validation; Writing – review & editing.

## CONFLICT OF INTEREST STATEMENT

The authors have declared that they have no competing or conflicts of interest.

## ETHICAL CONSIDERATIONS

Ethical approval was not required for this research review.

## Supporting information

Supporting Information S1

## Data Availability

No data is available except data in the manuscript and supplementary material.
