# Supplementary material for: Social gradient in use of health services and health‐related quality of life of children with attention‐deficit/hyperactivity disorder: A systematic review
Source: JCPP Adv. 2023 May 25;3(3):e12170. doi: 10.1002/jcv2.12170 (PMC10501702; doi:10.1002/jcv2.12170)
Supplement: Supplementary file 1 — Supporting Information S1 [file JCV2-3-e12170-s001.doc]

**Supporting Information**

**Appendix S1: Search terms included in systematic search**

| Search code | Search terms |
| --- | --- |
| S1 | child* OR adolescen* OR “young people” OR teen* OR youth* OR pediatri* OR paediatri* |
| S2 | “ADHD” OR “attention deficit hyperactivity disorder” OR “attention deficit disorder” OR “attention deficit disorder with hyperactivity” |
| S3 | Socioeconomic* OR “SES” OR “SEP” OR equit* OR inequit* OR "social disparit*" OR "social diff*" OR “social class” OR "class diff*" OR “economic class” OR economic disparit*" OR income |
| S4 | cost* OR (service* OR resource* OR “health care” OR healthcare) N2 (use OR utili*) OR “economic burden” |
| S5 | “wellbeing” OR well-being OR “well being” OR “personal satisfaction” OR “quality of life” OR perception OR QoL OR HRQoL OR utilit* |
| S6 | S1 AND S2 AND S3 AND S4 |
| S7 | S1 AND S2 AND S3 AND S5 |
| Limiters | Human, English, January 2010 - August 2022 |

**Appendix S2: Quality Assessment**
**Appendix 2.1: Critical Appraisal Skills Programme (CASP) Cohort study checklist**

| Author year | Cohort study question | | | | | | | | | | | | | |
| --- | --- | --- | --- | --- | --- | --- | --- | --- | --- | --- | --- | --- | --- | --- |
|  | 1 | 2 | 3 | 4 | 5 (a) | 5 (b) | 6 (a) | 6 (b) | 7 | 8 | 9 | 10 | 11 | 12 |
| D’Amico et al. 2014 | Y | Y | Y | Y | Y | Y | No (26% loss to follow up) | Y | The type of ADHD diagnosed, and socioeconomic factors were a predictor of long-term service costs as well as lifestyle outcomes. | High precision | Y | Y | Y | Yes, potential implications for planning and predictors of utility for children with ADHD. |
| Dejong et al. 2016 | Y | Y | Y | Y | Y | Y | Y | Can’t tell | Severity of symptoms and CSHCN screener items were associated with service expenditures. | High precision | Y | Y | Can’t tell | Yes, could influence planning and appropriate practice between diagnostic groups. |
| Enns et al. 2017 | Y | Y | Y | Y | Y | Y | Can’t tell | Can’t tell | Multimodal approaches to treatment lower inequity across the socioeconomic gradient. | High precision | Y | Y | Y | Yes, potential implications for planning and reduce inequity in service usage. |
| Jablonska et al. 2020 | Y | Y | Y | Y | Y | Y | Y | Can’t tell | Immigration status was found to be particularly associated with reduced treatment access. | High precision | Y | Y | Y | Yes, potential implications for planning and equitable service usage. |
| Laugesen et al. 2018 | Y | Y | Y | Y | Y | Y | Y | Y | The association between service usage was affected by an ADHD diagnosis, however explanatory causation could not be defined. | High precision | Y | Y | Y | Yes, potential implications for planning and appropriate practice. |
| Owens 2020 | Y | Y | Y | Y | Y | Y | Y | Y | There were many differences in HRQoL between SES groups and diagnosed/ non-ADHD children | High precision | Y | Y | Y | Yes, potential implications for planning and appropriate practice. |
| Sayal et al. 2015 | Y | Y | Y | Y | Y | Y | Y | Y | Under-privileged areas were weakly associated with higher service usage. | High precision | Y | Y | Y | Yes, potential implications for planning of service reach. |
| Ghosh et al. 2017 | Y | Y | Y | Y | Y | Y | Y | Y | Individuals from remote and disadvantaged backgrounds had stimulants at younger ages than individuals living in metropolitan areas and with least disadvantage. | High precision | Y | Y | Y | Yes, findings highlight the need for tailoring ADHD diagnosis, treatments and service delivery appropriately to children and adolescents from diverse cultures. |
| Kemper et al., 2013 | Y | Y | Y | Y | Can’t tell | Y | Y | Y | Household income, higher parental education were significantly associated with CAM therapy. | High precision | Y | Y | Y | Yes, this study supports further investigation on improving the access to care. |
| Bussing et al., 1998 | Y | Y | Can’t tell | Y | Can’t tell | N | Y | Y | poverty predicted lower treatment rates For Children with unmet needs for ADHD | High precision | Y | Y | Y | Yes, this study has health policy implications. |
| Bussing et al., 2003 | Y | Y | Y | Y | Can’t tell | Y | Y | Y | Low-SES Was negatively associated with visits to primary care provider. | High precision | Y | Y | Y | Yes, this study has health policy implications. |

*Notes: Y = yes*, *ADHD = Attention Deficit Hyperactivity Disorder,* CSHCN = Children with Special Health Care Needs Screening, CAM = *Complementary and Alternative Medical therapies, SES = Socioeconomic Status. (from https://casp-uk.net/casp-tools-checklists/)*

**Appendix 2.2: The Joanna Briggs Institute (JBI) Cross-sectional study checklist**

| Author year | JBI Cross sectional study questions | | | | | | | | |
| --- | --- | --- | --- | --- | --- | --- | --- | --- | --- |
|  | 1 | 2 | 3 | 4 | 5 | 6 | 7 | 8 | Overall appraisal |
| Nasol et al. 2019 | Yes | Yes | Yes | Yes | Yes | Yes | Yes | Yes | Included |
| Ronis et al. 2015 | Yes | Yes | Yes | Yes | Yes | Yes | Yes | Yes | Included |
| Tzang et al. 2014 | Yes | Yes | Yes | Yes | Yes | Yes | Yes | Yes | Included |
| Van Der Kolk et al. 2014 | Yes | Yes | Yes | Yes | Yes | Yes | Yes | Yes | Included |
| Stevens et al., 2005 | Yes | Yes | Yes | Unclear | Yes | Unclear | Yes | Yes | Included |
| Cuffe et al., 2009 | Yes | Yes | Yes | Yes | Unclear | Unclear | Yes | Yes | Included |
| Toomey et al., 2012 | Yes | Yes | Yes | Yes | Yes | Yes | Yes | Yes | Included |
| Kendall et al., 2005 | Yes | Yes | Yes | Yes | Yes | Unclear | Yes | Yes | Included |

*Note: JBI = The Joanna Briggs Institute (from https://jbi.global/critical-appraisal-tools ). Lee et al., 2022 was not assessed as it was a comparative study and authors claimed that it was not a cross-sectional design*

**Appendix 2.3: Critical Appraisal Skills Programme (CASP) Literature Review Checklist**

| Author year | Literature review Questions | | | | | | | | | |
| --- | --- | --- | --- | --- | --- | --- | --- | --- | --- | --- |
|  | 1 | 2 | 3 | 4 | 5 | 6 | 7 | 8 | 9 | 10 |
| Lindly et al. 2021 | Yes | Yes | Can't tell | No | Yes | An update on the results of recent studies regarding disparities in SES and ADHD towards equitable outcomes for children with ADHD. | Can’t tell | Yes | Yes | Yes |
| Green & Langburg 2021 | Yes | Yes | Yes | No | Yes | This review synthesizes evidence on predictors of ADHD psychosocial intervention utilization in clinic, community, and school settings | Can’t tell | Yes | Yes | Yes |
| Wright et al. 2015 | Yes | Yes | Yes | No | Yes | Low socioeconomic status was identified as an indicator of unmet need, approximately doubling the odds that a child with ADHD would not receive services | Precise enough | Yes | Yes | Yes |

*Note: ADHD = Attention Deficit Hyperactivity Disorder, SES = Socioeconomic Status (from https://casp-uk.net/casp-tools-checklists/)*

**Appendix 2.4: The Mixed Methods Appraisal Tool (MMAT) Checklist**

| Author year | MMAT questions | | | | | | |
| --- | --- | --- | --- | --- | --- | --- | --- |
|  | S1 | S2 | 5.1 | 5.2 | 5.3 | 5.4 | 5.5 |
| Laugesen et al. 2020 | Yes | Yes | Yes | Yes | Yes | No | Yes |

*Note: Section 5 of the MMAT Checklist was applicable for mixed method studies (from http://mixedmethodsappraisaltoolpublic.pbworks.com/w/file/fetch/127916259/MMAT_2018_criteria-manual_2018-08-01_ENG.pdf)*

**Appendix 2.5: Critical Appraisal Skills Programme (CASP) randomized controlled trials (RCT) study checklist**

| **Author year** | **RCT questions** | | | | | | | | | | |
| --- | --- | --- | --- | --- | --- | --- | --- | --- | --- | --- | --- |
|  | **1** | **2** | **3** | **4** | **5** | **6** | **7** | **8** | **9** | **10** | **11** |
| Kamimura et al., 2022 | Yes | Yes | Yes | Yes, Yes, Can’t tell | Yes | Can’t tell | Yes | Yes | No | Yes | Can’t tell |

*(From* *https://casp-uk.net/casp-tools-checklists/)*
